# How rankings disguise gender inequality: A comparative analysis of cross-country gender equality rankings based on adjusted wage gaps

**DOI:** 10.1371/journal.pone.0241107

**Published:** 2020-11-04

**Authors:** Karolina Goraus Tanska, Joanna Tyrowicz, Lucas Augusto van der Velde

**Affiliations:** 1 Faculty of Economic Sciences, University of Warsaw, Warsaw, Poland; 2 FAME|GRAPE, Warsaw, Poland; 3 Faculty of Management, University of Warsaw, Warsaw, Poland; 4 IZA, Institute of Labor Economics, Bonn, Germany; 5 Institute of Statistics and Demographics, Warsaw School of Economics, Warsaw, Poland; University of Reading, UNITED KINGDOM

## Abstract

Methods for estimating the scope of unjustified inequality differ in their sensitivity to address institutional and structural deficiencies. In the case of gender wage gaps, adjusting adequately for individual characteristics requires prior assessment of several important deficiencies, primarily whether a given labor market is characterized by gendered selection into employment, gendered segmentation and whether these mechanisms differ along the distribution of wages. Given that countries are characterized by differentiated prevalence of these deficiencies, ranking countries on gender wage gaps is a challenging task. Whether a country is perceived as more equal than others depends on the interaction between the method of adjusting gender wage gap for individual characteristics and the prevalence of these deficiencies. We make the case that this interaction is empirically relevant by comparing the country rankings for the adjusted gender wage gap among 23 EU countries. In this relatively homogeneous group of countries, the interaction between method and underlying deficiencies leads to substantial variation in the extent of unjustified inequality. A country may change its place in the ranking by as much as ten positions–both towards greater equality and towards greater inequality. We also show that, if explored properly, this variability can yield valuable policy insights: changes in the ranking positions across methods inform on the policy priority of the labor market deficiencies across countries in relative terms.

## 1. Introduction

In this paper, we study cross-country rankings of gender inequality. For many aspects of gender inequality, policy debates focus on cross-country rankings. The rankings are obtained by specialized institutions, who first estimate levels of inequality and subsequently rank countries on one such measure or construct a composite index of such measures. Utilized for both policy-evaluation and policy-making purposes (for Sweden and Switzerland see [1, 2], Bloomberg systematically ranks the US states for gender equality [3]), the rankings are used to shape the public debate, set policy objectives and deploy public funding. Indeed, the debate on gender equality is largely influenced by cross-country rankings, see, for example [4–6]. In particular, after the World Economic Forum (WEF) published The Global Gender Gap Report in 2018, the US was shamed for ranking 49th in the world on talk-shows and in print [7], Japan was shamed in the media for ranking the worst among G7 countries [8] and The Philippines were praised for ranking as the most equal among South-East Asian countries [9]. In a similar spirit, Forbes pursued with coverage of top-ranked countries, naming a few policies that were deemed relevant for achieving high levels of gender equality (even though the listed policies and the structure of the WEF gender equality index were not actually related [10]). In the European Union, the publication of rankings in gender wage equality based on harmonized linked employer-employee data every four years attracts coverage from the European Commission, national governments, and media alike.

However, in order to rank the countries, one first has to obtain the measures of inequality, thus inevitably facing the choice of a proper measure of inequality. In academia, there appears to be a broad consensus that comparisons of economic outcomes across genders should be adjusted for differences in the underlying characteristics–a process referred to as decomposition–even though most of the publicly debated rankings are based on raw differentials. Two reasons explain the prevalence of raw differentials in the public debate. First, in order to adjust the gender wage gaps for differences in the underlying characteristics, one requires access to individual-level data, while most of the global and regional rankings are based on readily available aggregates. Second, while academics consistently emphasize the paramount relevance of adjusting the measurement of gaps in outcomes for differences in individual characteristics, there is no consensus on the choice of the specific decomposition method. Recent decades have seen a growing proliferation of methods, data sources, and model specifications, reaching effectively hundreds of potential combinations between data sources, set of control variables, and decomposition method. As formally discussed by [11], depending on the underlying process of wage formation, labor market segmentation, and the causes behind the wage gaps, different estimators perform with varying reliability. For example [12], demonstrate a remarkable dispersion of gender wage gap estimates for one data source for one country in one year, obtaining roughly 2500 estimates of the adjusted gender wage gap. The multiplicity of gender wage gap estimates stems from systematically manipulating control variables and methods, and the estimates differ by more than 100% of the lowest obtained estimate.

The dispersion between the obtained estimates stems from the fact that each method differs in its ability to reflect various labor market phenomena–or deficiencies. For example, some methods operate by design at the mean of the income distribution (such as parametric regression-based methods), and thus, they do not adequately reflect the scope of unjust inequality if sticky floors or glass ceilings are important in a given labor market. The problem is all the more acute for international comparisons because labor market deficiencies can differ across countries, making specific methods suitable for some countries, but not for the others. For example [13], demonstrated the substantial differentiation worldwide of the estimated inequality measures adjusted for differences in individual characteristics: their estimates for 63 countries range from 8% to as much as 48% of female income (in one given year). However, while using one method for all the countries makes the estimated wage gaps comparable, it makes the estimates possibly ill-suited for some countries, undermining the validity of ranking them according to this measure of inequality. One can extend this argument to any other decomposition method.

In this paper, we illustrate that country rankings of gender wage inequality differ substantially depending on the methodological choice. We show that a country can change its ranking by roughly ten places towards greater equality or greater inequality, depending on whether the underlying decomposition method accounts for selection into full-time employment or not. These results quantitatively corroborate the concerns about the reliability of cross-country rankings.

Further, we illustrate that the changes in the gender wage inequality rankings are systematically related to labor market features. We also show that changes in rankings across specifications correlate well with the measures of working time flexibility and work-life balance. These findings illustrate that the cross-country rankings may vary systematically with the interaction between the method of estimation and the institutional features of the labor markets.

In order to deliver these results, we utilize individual-level data across 23 EU countries. We purposefully selected data sources harmonized in terms of sample design, questionnaire, and implementation, in order to limit the role of data idiosyncrasies in cross-country rankings. We apply the decomposition techniques introduced in prior literature (see [11, 12]) to these data sets and obtain estimates of adjusted gender wage gaps, to which we refer as measures of *unjustified inequality*. We subsequently rank countries on those measures and study the links between rankings, methodological choices, and labor market deficiencies.

The paper is structured as follows. We first discuss state of the art on (gender) wage gaps measurement and demonstrate the relevance of methodological choices for cross-country comparisons in section 2. Based on this overview, we describe our data and methods in section 3. The results are reported in section 4. The concluding remarks and policy implications summarize our study.

## 2. Measuring the wage gaps in the international context

Consider a population consisting of Robinson Crusoe and Friday: the two individuals are highly differentiated in skills (individual characteristics) and caloric intake (outcome). The differences in caloric intake partly stem from relevant and observable skills (e.g., survival skills), irrelevant and observable skills (e.g., literacy and knowledge of contemporaneous literature), and policies, which in this simple example are represented by social structure imposed by Crusoe on Friday. Raw differences in caloric intake effectively underestimate the scope of inequality of outcomes in that population. While the measure of caloric intake is objective, it is not (fully) informative of the extent of *unjustified inequality* in outcomes. To grasp the *unjustified inequality*, one has to account for differences in outcome-relevant characteristics.

**Fig 1:**
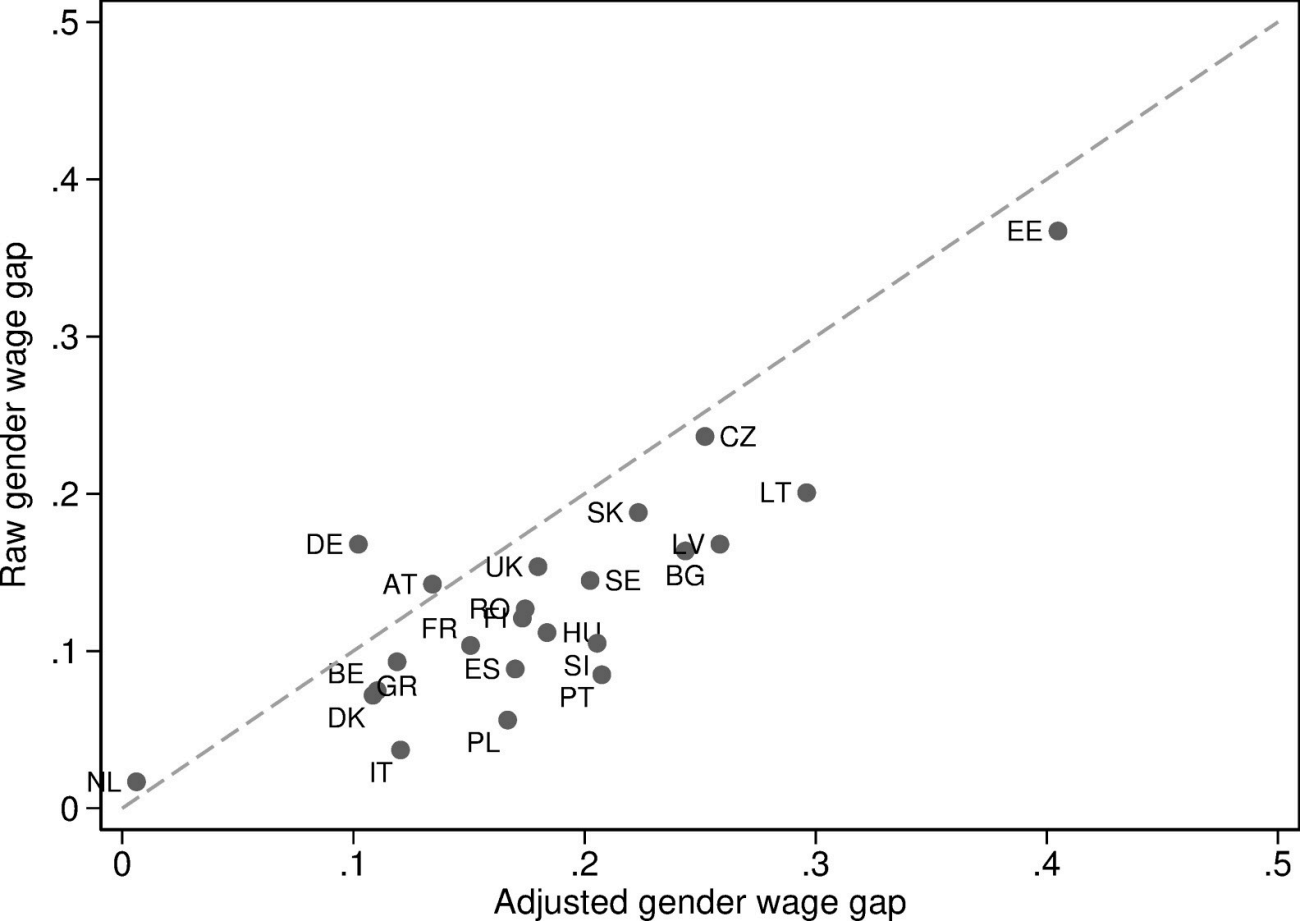
Raw vs. adjusted gender wage gaps. Data details described in section 3. The raw wage gap computed as $\overset{-}{w_{m}}-\overset{-}{w_{f}}$. The adjusted gender wage gap computed from Oaxaca-Blinder decomposition with the following set of controls: age, education, residence, and marital status. Full set of estimates is discussed in section 4. Estimates obtained separately for each country. The dotted line represents a 45 degree line.

As means of motivation, Fig 1 reports the size of the raw gender wage gap (as published regularly by the Eurostat and used by both media and policymakers to identify the scope of gender-related inequality across the EU member states) and the estimates of adjusted gender wage gap (AGWG) using the most common decomposition method. If the raw wage gap was indicative of the actual scope of unjust wage inequality, the countries should be located along the diagonal in Fig 1, which is clearly not the case. The estimated adjusted wage gaps differ by as much as 20 percentage points from the raw gender wage gap.

### 2.1. Decomposition methods to uncover *unjustified inequality*

To account for objective drivers of wage dispersion–as opposed to merely raw inequality–academic research relies on methods that *adjust* differences in outcomes for differences in outcome-relevant characteristics. Following [14] and [15], parametric techniques decompose observed differences in outcomes (raw wage gaps) into two components: differences in the underlying characteristics and differences in how these characteristics matter in defining outcomes. In the case of gender wage gaps, this is obtained by estimating, at least, two separate wage regressions. The size of the pay difference can be decomposed into *W*_*m*_ − *W*_*f*_ = *β*^∗^(*X*_*m*_ − *X*_*f*_) + *X*_*m*_(*β*_*m*_ − *β*^∗^) + *X*_*f*_ (*β*^∗^ − *β*_*f*_), where the *W*_*m*_ − *W*_*f*_ stands for the unadjusted (or raw) gender wage gap (GWG), (*X*_*m*_ − *X*_*f*_) represents differences in characteristics between men and women, and (*β*_*m*_ − *β*^∗^) and (*β*^∗^ − *β*_*f*_) stand for the differences in coefficients related to male and female (dis)advantages, respectively. The literature refers to this last term as an adjusted gender wage gap (AGWG).

For a few decades, the literature on the gaps in wages, education, etc.–has been dominated by a handful of techniques, namely the [14] and [15] decomposition, with the extensions for functional form proposed by, e.g. [16–20]. These estimates were referred to as adjusted wage gap, i.e., the wage gap that remains after adjusting for differences in characteristics important for productivity (such as age, education, industry, occupation, firm characteristics, etc.). The parametric Oaxaca-Blinder decomposition assumes that in the absence of discrimination, the disadvantaged group would record earnings according to the advantaged group (counterfactual wage structure, see [14, 15]). In the case of gender, this is equivalent to setting *β*^∗^ = *β*_*m*_. However, alternative assumptions are possible, affecting the interpretation of the adjusted gap. If one believes that the advantaged group receives a premium, one can set β∗ = βf to be the fair wage structure [16]. suggested to use simple average of coefficients in both groups, then *β*^∗^ = 0.5*β*_*m*_ + 0.5*β*_*f*_ [20], recommended weighted average, *β*^∗^ = %*men*∗*β*_*m*_ + %*women*∗*β*_*f*_. Studying the algebraic properties of this estimator [21], demonstrated that the weights should be the opposite, but this differentiation is less relevant if shares of men and women in the labor market are fairly comparable. Finally, for interpretative ease [17], proposed to use the coefficients from pooled regression without gender dummy and [22] from pooled regression with a gender dummy.

Regardless of the assumptions behind the counterfactual wage structure, these methods share a common feature: they derive the scope of unjust (unexplained by individual characteristics) wage inequality from parameters estimated at the mean, and hence they provide little information of inequality at different points of the outcome distribution.

This approach is troubled with weaknesses already acknowledged in the literature. First, if the analyzed subpopulations of men and women differ by characteristics (e.g., women are better educated; or jobs are segregated across genders), the parametric decomposition at the mean may be meaningless: not a single man or woman could be "similar" to the sample mean. Second, the parametric regression-based approaches cannot account for the sticky floors or glass ceilings (e.g., due to differentiated access to top paying positions). Third, if the patterns of selection into e.g., employment differ across genders, the parametric decompositions assign to wage mechanisms what is effectively unrelated to wages *per se* but is related to employment. All three of these problems may generate a significant bias in the results for wage gaps, and analogous examples can be established for other outcome measures.

A wide array of new methods addresses one or more of these shortcomings. For example, the parametric decomposition methods have been extended to allow for selection into employment a la [23], with specificity of the selection patterns translating directly to the measures of unjust inequality. There was substantial effort into providing decomposition methods for non- continuous outcomes (e.g. self-employment, access to public service, health status [24, 25]. There are also many semi-parametric or non-parametric methods, whose major advantage is that they allow to go beyond the mean and analyze continuous outcomes along their distributions [26–28]. Again, accounting for selection into employment is a challenge, addressed partially, see [29, 30]. The urge to compare only the comparable implies that a decision needs to be made about what “comparable” actually means [31]. proposes an exact matching method to identify “comparable” individuals and then isolate the "incomparable" individuals in both groups to infer the possible selectivity in the wage process. An alternative approach consists of reweighing the distribution of one group to replicate the distribution of the other group in terms of individual characteristics [32].

The literature provides a wide selection of methods to obtain measures of AGWG which also operate along with the wage distribution rather than simply at the mean [26–28, 32]. While estimating the AGWG with this technique, any parametric decomposition may be applied for obtaining the parameters of the wage equation, adjusted for the distributional properties of wages. The methods that rely on the functional form of the estimated wage regression may lead to issues if the model is misspecified and model parameters are biased. The literature also provides semi-parametric and non-parametric alternatives to estimate AGWG. In [32], the counterfactual conditional distribution is obtained via a reweighing procedure through which the attributes observed among women (men) are given weights such that the resulting distribution of characteristics resembles that of men (women). Hence, this technique utilizes information about the similarity of both male and female populations in terms of underlying characteristics relevant to wages. The non-parametric decomposition proposed by [31] applies exact matching to construct a counterfactual population of women. The advantages of this decomposition method comprise (a) comparing the comparable because prior to matching, a common support restriction is applied, and (b) the validity of the estimates does not rest on functional form assumptions. Moreover, differences between workers inside and outside of the common support are informative of the consequences of segmentation.

Despite the exceptional advancement in this field, challenges remain. Ideally, one would want to compare individuals who are actually "alike" in terms of all relevant characteristics other than gender. In the case of gender wage gaps, such ideal comparison should include hours effectively worked, commitment, talent, etc. Unfortunately, many of these characteristics are not observable or are imperfectly measured, e.g., human capital (cfr. discussion in [11]). Moreover, one would ideally want the world to exhibit only selected dimensions of inequality: e.g., gendered employment selection and, additionally, gender gaps in wages. In such a case, there is a suitable decomposition method for obtaining reliable measures of unjust inequality. However, with gendered employment selection combined with gender sorting of workers and gender wage gaps differentiated across the distribution of income–then no single decomposition method can adequately account for unjust inequality in wages across genders. If, furthermore, we want to obtain ranking of countries on gender wage gaps and countries may differ in which deficiencies they exhibit and to what extent–then we face a nearly impossible task.

### 2.2. Ranking unjustified inequality

In international comparisons, one often relies on rankings rather than actual measures. This is particularly true in the case of gender inequality. Adjusted wage gaps are not intuitive in interpretation, furthermore, wage equality is by far not the only issue raised in public debate: WEF report includes measures of health issues, access to education, etc.; European Gender Equality Index (EGEI) and the Social Institutions and Gender Index also includes political freedoms, experience of violence, etc. (see [33, 34]) Composite indices, which account for many dimensions of inequality, are even less intuitive in interpretation, which implies intensifying use of rankings for international comparisons. Given this complexity, rankings are developed, e.g., across US states [35], or across countries [4–6]. The rankings could be misguiding if they are based on measures that are suitable for capturing inequality in some countries, but not in others (Box 1).

**Box 1. An illustrative example**

Consider a following stylized example. There are four countries (A, B, C and D). Country A has equal wages across workers on a given job, but access to jobs is highly segmented across genders: men occupy high paying positions and women occupy low paying positions. Country B has equal access to jobs, but primary care givers have no access to institutionalized care. In this country, due to caring, women are considered as less reliable workers irrespective of their individual abilities (statistical discrimination), hence wages are unequal across genders for the same job. In the final two countries, caring facilities are available, and labor market is not segmented, but country C is characterized by pure taste based discrimination of women, whereas country D is not. For the sake of simplicity, assume that raw wage differences *among workers* in these four countries are roughly equal, so that a ranking based on raw wage differences is not very informative. Further, assume men and women in these countries have the same distribution of productivity-relevant characteristics.

- Decomposition methods sensitive to gendered employment selection patterns and insensitive to whether or not actually similar workers are compared will rank B as the most unequal country, subsequently ranking C and A as giving unequal pay for equal work (i.e. confusing segmentation for unjustified wage inequality).
- Some decomposition methods are both sensitive to gendered selection and adjust for comparing only similar workers. These methods will rank countries C and A differently.
- Decomposition methods sensitive to segmentation will rank A as the most unequal, followed by B, whereas C and D will be considered equal.

Comparing just these three rankings, once their sensitivity to given deficiencies relative across genders are understood, one can infer the relative relevance of the deficiencies from the changes in the ranking between A, B, C and D. Exploring differences across rankings will not be relevant for D, but will be informative to specify the priorities in policy interventions for the other three countries.

**Fig 2:**
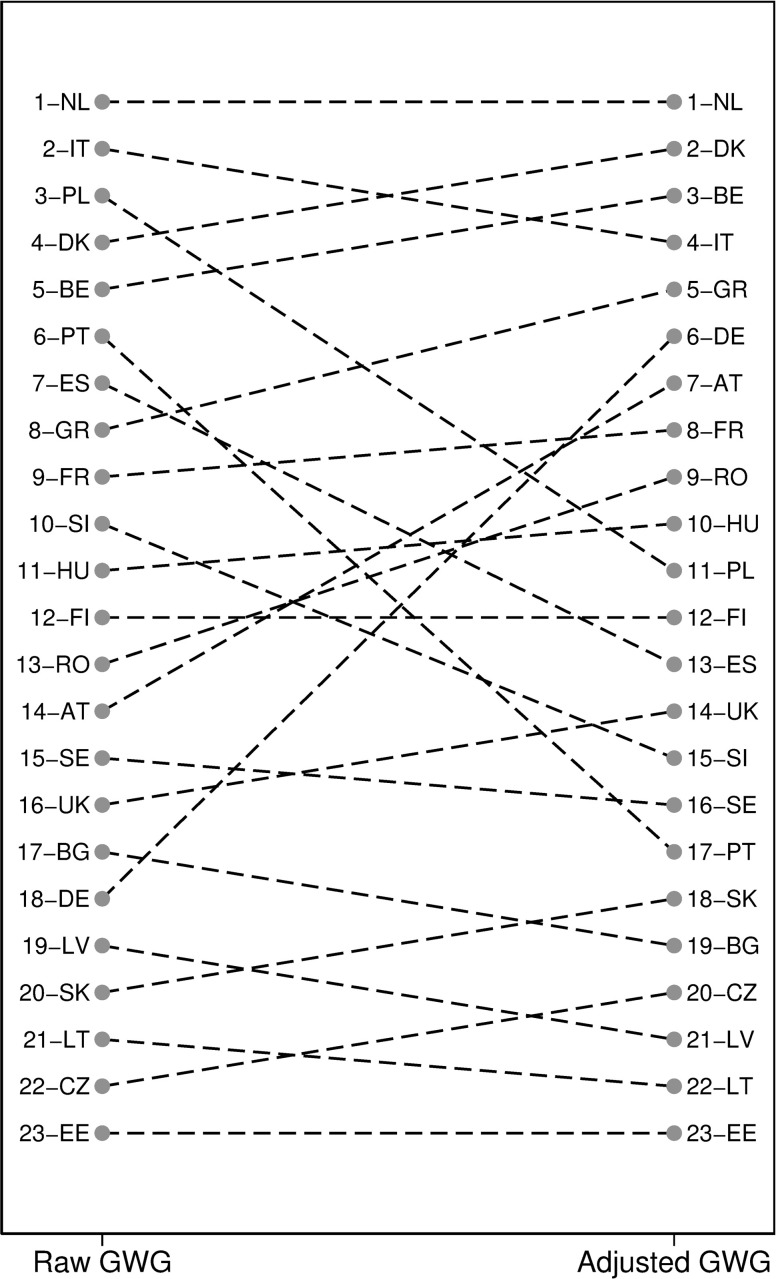
Changes in the ranking of countries: Raw and adjusted GWG. A lower rank identifies countries with lower inequality. Ranking based on raw gap measures and adjusted gap estimates, see the note under Fig 1 for details.

In Fig 2, we portray the same data as in Fig 1, but in the form of rankings rather than actual measured levels of inequality. Countries like Germany move 14 ranks up towards greater equality, whereas Poland or Portugal both rank 11 positions lower towards greater inequality when considering adjusted gender wage gaps rather than raw wage gaps.

Recall that according to Fig 1 in all but two countries, inequality grows when we change the metric of gender wage gaps from raw to adjusted. However, this increase in inequality is differential across countries: it does not preserve the ranking of countries. This simple observation implies that both the differences in characteristics of men and women in the labor market and the scope for how unequal pay women receive for an equal job differ across European countries. In some countries, differences in characteristics between men and women are responsible for a relatively more significant share of the wage gap than in others. In other words, unjust inequality is in fact, a more pressing problem in some countries (Poland, Portugal) than what could infer by a ranking based on raw wages. The converse is true for other countries, such as Germany, where a relatively large part of gender differences are due to objective differences in workers' characteristics rather than how these characteristics are unjustly rewarded. Consequently, comparing the rankings on raw and adjusted gender wage gaps reveals which type of inequality–differences in characteristics between men and women or differences in how the same characteristics are rewarded–is more prevalent in a given country in a comparative (relative) perspective.

Studying Fig 2 reveals that comparing rankings across methods for a given set of countries can help establish policy priorities. If a ranking position of a country worsens substantially between the decomposition methods, then this country is relatively more troubled by a deficiency to which a given method (used to obtain measures underlying the rankings) is more sensitive. As mentioned, higher rankings represent more unjustified inequality.

In the remainder of the paper, we utilize the decomposition methods developed in prior literature to provide comparable estimates of the adjusted gender wage gaps. We obtain estimates at means and along with the distribution of wages, applying parametric, semi-parametric, and non-parametric methods. We also vary the set of covariates used in obtaining the adjusted wages, expanding from a basic set (age, education, residence, and family situation) to account for gender sorting (across occupations and industries) and firm characteristics. We subsequently utilize these estimates to rank countries and study these rankings to uncover the factors that lead to the largest changes in the ranking across countries. Finally, we show how those changes in rankings are related to the deficiencies of the respective labor markets.

## 3. Data and methods

We focus on countries from the European Union (EU), since these countries embarked on efforts to harmonize policies addressing inequality. Focusing on EU countries has several advantages over alternative groupings: these countries have a relatively similar level of development; they provide harmonized data; their borders are open for labor flows, and they differ in labor market imperfections affecting selection into employment and wages.

Given differences in data collection and definitions across countries, estimating meaningful measures of unjustified inequality for international comparisons constitutes a challenge on its own. Even in the EU, which leads plausibly the most comprehensive effort to harmonize individual data, there is no source of earnings database that would permit a comprehensive comparison of wage gaps. We use data from the European Union Survey of Income and Living Conditions (EU-SILC) covering 23 countries for the year 2013. EU-SILC is a rich source of information about worker and job characteristics. In addition to individual characteristics (age, gender, education, marital status and controls for urban density at current location residence) it has a large number of job-related characteristics: occupation (one digit ISCO), industry (one digit NACE) and firm-size (under ten workers, 11–49 workers and above 50 workers). Moreover, the database contains workers in all types of companies, be it private or state-owned. Given this richness, we can estimate the adjusted gender wage gap (AGWG) for various sets of controls: individual characteristics, augmented by occupation, industry, and firm-size.

All in all, we study 23 countries, as reported in Table 1, along with the sample sizes. The 23 countries in the study meet the following criteria: they are EU member states at the moment of survey, they have common reference period for reporting income and labor market status, and the sample size is large enough. Data for Croatia, Iceland, Norway, Turkey, FYROM, and Switzerland do not meet the first criterion. Data for Ireland fails to meet the second criterion. Data for Cyprus, Luxembourg and Malta do not meet the third criterion.

**Table 1:** Data availability per country.

Country	N	% age 18–65	% employed	% one job	% FTFY	% wage available	% complete observations
AT	10940	0.75	0.63	0.60	0.29	0.27	0.24
BE	11711	0.76	0.61	0.60	0.27	0.27	0.24
BG	10880	0.71	0.59	0.59	0.33	0.33	0.27
CH	14034	0.73	0.58	0.53	0.23	0.23	0.21
CY	10988	0.76	0.59	0.58	0.34	0.34	0.28
CZ	16275	0.74	0.62	0.61	0.37	0.37	0.36
DE	22585	0.72	0.66	0.62	0.30	0.30	0.28
DK	10982	0.73	0.63	0.59	0.36	0.36	0.16
EE	12551	0.72	0.62	0.60	0.33	0.33	0.30
ES	26883	0.75	0.56	0.56	0.24	0.24	0.21
FI	22486	0.76	0.57	0.55	0.29	0.29	0.13
FR	20984	0.74	0.61	0.60	0.32	0.32	0.25
GR	15318	0.70	0.40	0.39	0.16	0.16	0.14
HR	12218	0.71	0.56	0.55	0.25	0.25	0.20
HU	21349	0.77	0.63	0.62	0.33	0.33	0.29
IS	6943	0.78	0.65	0.60	0.35	0.35	0.14
IT	38039	0.73	0.48	0.47	0.24	0.24	0.22
LT	10485	0.68	0.58	0.55	0.31	0.30	0.27
LU	8005	0.81	0.70	0.69	0.36	0.35	0.31
LV	12442	0.70	0.59	0.57	0.31	0.31	0.27
MT	10201	0.73	0.57	0.55	0.31	0.31	0.30
NL	19476	0.79	0.67	0.64	0.24	0.24	0.10
NO	11998	0.77	0.68	0.62	0.40	0.40	0.19
PL	30162	0.71	0.54	0.51	0.26	0.26	0.23
PT	14009	0.71	0.57	0.56	0.31	0.31	0.26
RO	15859	0.72	0.47	0.45	0.29	0.29	0.25
RS	16967	0.76	0.48	0.46	0.23	0.23	0.20
SE	12223	0.71	0.61	0.57	0.33	0.33	0.15
SI	23374	0.78	0.62	0.61	0.37	0.37	0.33
SK	13286	0.79	0.64	0.64	0.40	0.40	0.36
UK	18336	0.74	0.62	0.61	0.33	0.31	0.27

For responders who report holding more than one job, identifying earnings from each job separately is impossible. FTFY denotes full-time full year employment. Data on individual earned income are available on a yearly basis. While most variables in the EU-SILC reflect the current situation of the surveyed individuals, the information on income relates mainly to the previous calendar year. Thus, the EU-SILC data from 2013 in most cases provide information on incomes from 2012. We name the data points according to the reference period of the income information; PL 2012 refers to data from the EU-SILC 2013 round in Poland, while UK 2012 refers to the EU-SILC 2012 round in the United Kingdom. We also exclude Ireland from the analysis, as the income reference periods may not overlap for women and men.

To have an objectively comparable metric, we study hourly wages of full-time full-year employees who hold one job at a time and who did not change jobs within that year (sample denoted as FTFY). In the most prevalent way to report incomes in EU SILC, individuals report the total labor income earned in the previous year. Individuals also report the number of hours typically worked in a week prior to the study. To obtain comparable measures of income, we need to convert the annual incomes to average hourly rates, which is only possible in a meaningful way only for full-time full-year employees. First, we cannot use data for part-time workers, as the period used for reporting hours does not overlap with the period for reporting income and the actual number of hours worked might vary more than among full time workers, hence imputing hourly wage would introduce additional sources of variance, which may be systematically related to gender or other individual characteristics. Second, individuals do report the number of months worked, but report hours only in the past week. Hence, for individuals who did not work the full year, there is no data on the total number of hours. Third, we cannot use the data for workers whose situation changed (e.g., changed jobs, held a job with different number of hours, etc.). Consequently, we obtain comparable estimates of the AGWG from the sample of full-time and full-year employees (FTFY). In Table 1, we report the coverage of this metric across countries.

While the use of FTFY workers may seem like a limitation, it actually serves to identify the differences in access to employment opportunities. In a country where few women work FTFY, the adjusted gender wage gaps obtained only for those workers may show relatively equal wages if the majority of the gender penalty is due to a part-time penalty. In this case, the institutional features of these labor markets reflect an important aspect of gender inequality, which is reflected in selection into FTFY employment rather than wages per se, see [36–39]. Indeed, the incidence of part-time employment and unemployment, the role of selection into FTFY employment, can inform about the role of segmentation between FTFY and irregular jobs in the adjusted gender wage inequality.

Also, the ability to manipulate the set of control variables is particularly desirable if some labor markets are more segregated on gender than others. Methods that do not adjust for segregation yield higher estimates of AGWG (in relative terms) for countries characterized by highly segmented labor markets, *ceteris paribus*. Hence, comparing rankings based on AGWG estimates with and without an occupation or industry characteristics can reveal the extent to which segregation stands behind gender inequality in comparative terms.

We explore all combinations of methods and covariates. First, among parametric estimates at the mean, we included all possible combinations of male-female coefficients discussed in the literature [14–17, 20–22]. This yields seven variants. Moreover, we estimated these decompositions, correcting for gendered selection into FTFY employment (*a la Heckman*), which doubles the number of variants (with and without selection adjustment) The Heckman correction was not applied to the methods proposed in [17, 22] as these approaches require estimating an additional regression for obtaining the counterfactual wage structure. Selection correction [23] for selection into employment uses as exclusion criterion the information on the household structure (e.g., presence of children under the age of 6 in the household) and alternative sources of income (e.g., presence of another earner in the household and the availability of non-labor income in the household). Further, all estimates are obtained on a sample restricted to observations for which a statistical twin of the opposite gender is observed and without this restriction. A subset of the estimations contains both selection correction term and a common support restriction, in this case, the common support requires that we observe an individual with the same characteristics in each combination of the two dichotomies: FTFY or not, and men or women. This raises the number of variants further by a factor of two.

For other methods, the range of combinations is smaller. Non-parametric models lack an equivalent of a selection correction model a la [23]. Then, and by definition, the Nopo decomposition can only be estimated within the common support [31]. For semi-parametric models, we consider the reweighting scheme described in [32].

Outside of the mean, we estimate decompositions at the quartiles (25th, 50th and 75th percentiles). We include the parametric decomposition of [26], and utilize the semi-parametric methods of [28] and [32]. The first two methods allow changing the non-discriminatory wage structure in the same way as parametric decompositions at the mean, and so our estimates vary along this dimension. To the best of our knowledge, no procedure incorporates a selection correction into these models. In these models, we opted to exclude common support correction given that it is not clear whether the restriction should apply to the specific unconditional quartile or to the entire sample.

Method-wise, we obtain between 7 and 28 variants at the mean and 7 variants at each quartile. The detailed account for all methods is summarized in Table 2. For each method, we estimate AGWG using alternative sets of conditioning variable. All estimations account for the “basic” set of demographic and human capital variables. In this set, we include controls for age, education, marital status, and the degree of urbanization in the place of residence. We then expand step-wise the conditioning set to include industry, firm size controls, and occupation controls. This yields 189 variants at the mean for each country and 91 variants at each of three quartiles for each country.

**Table 2:** Account of the decomposition methods used in this study.

Method	Number of specifications				
	Counterfactual wage structure	Selection	Common support	Covariates	Total
**At mean**					
Parametric with selection [14–16, 20, 21]	5	2 (Yes & No)	2 (Yes & No)	7	140
Parametric without selection [17, 22]	2	1 (No)	2 (Yes & No)	7	28
Semi-parametric (reweighting) [32]	1	1 (No)	2 (Yes & No)	7	14
Non-parametric [31]	1	1 (No)	1 (Yes)	7	7
Total					189
**At quartiles (25**^**th**^**, 50**^**th**^ **& 75**^**th**^ **percentile)**					
Parametric [26]	5	1	1	7	35
Reweighting [32]	1	1	1	7	7
RIF [28]	7	1	1	7	49
Total					91

Parametric refers to linear decompositions at the mean. Parametric with selection refers those parametric methods which allow for selection correction, these are the contributions of [14–16, 20, 21]. Parametric without selection refers to those parametric methods for which additional regression has to be estimated and selection into FTFY cannot be corrected, these are the contributions of [17, 22], which require the estimation of additional regressions. The remaining rows refer to the decompositions in parentheses DFL [32], Nopo [31], JMP [26] and RIF [28]. Seven potential sets of covariates includes: basic set, with occupation, with broad industries, with industry at 1 digit, with occupation and broad industries, with occupation and industry at 1 digit, with firm size. The total column is the product of the preceding columns and indicates the number of estimations per decomposition type.

The estimations of AGWG are performed for each country separately. In sum, for each country we obtain a total of 189 estimates of the AGWG at the mean and 91 estimates at each quartile. We use log of hourly wages as dependent variable, except [31] decomposition which operates in levels and reports results in percent. After we obtain estimates of the AGWG for each country using every possible specification (methods × control variables × mean/quartiles), we rank all countries under each specification. A lower rank identifies countries with lower AGWG.

## 4. Results

**Fig 3:**
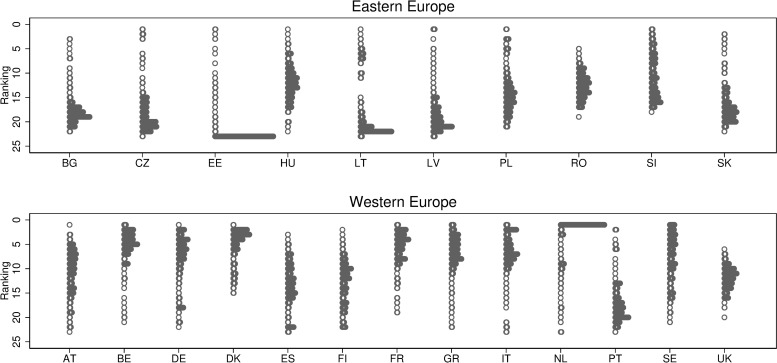
The distribution of rankings across countries. For each country we report a complete set of rankings, for each of the 462 methods applied. Each circle portrays a separate model. The dotplot portrays the concentration of rankings. Detailed data available at [http://grape.org.pl/data/gender-wage-gaps-around-eu-and-across-methods].

Fig 3 documents substantial dispersion in the ranking position of 23 EU countries in terms of adjusted gender wage inequality. There are just five countries in the sample that never rank 20 or worse (the lowest ranking for Denmark is 11, the lowest ranking for Slovenia is 18, and the lowest ranking for France and Romania is 19). Also, just two countries never rank ^fifth^ or better (best ranking for the United Kingdom is sixth6 and for Romania is 5). Even Estonia, a country that is often found at the bottom of the ranking, displays the lowest unjustified in some specifications. This considerable dispersion is achieved on the most comparable data set for the EU countries, i.e., EU-SILC.

We argue that this dispersion is informative in a sense that it can be used to infer in relative terms the severity of a given driver of *unjustified* gender inequality in wages. We formulate indirect inference: an issue a is more pressing in country j if country j moves to a worse ranking whenever we use a method which is sensitive to a. Specifically, we demonstrate that methods and labor market deficiencies are related across countries such that if a given deficiency is more prevalent in one country than in others, the ranking of this country worsens relative to others in a ranking based on a method sensitive to a given type of deficiency.

**Fig 4:**
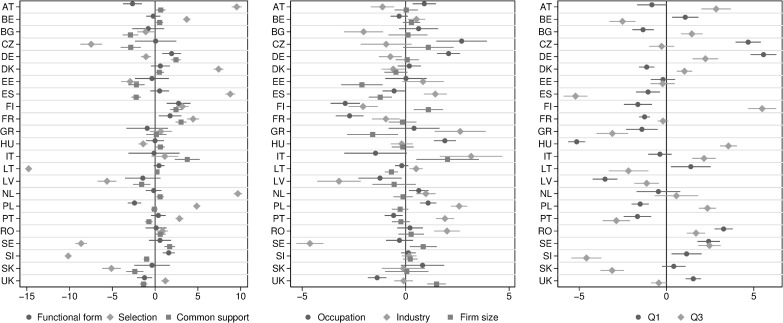
Ranking of countries and method of estimating AGWG. The horizontal axis denotes the number of positions in the ranking. Negative numbers signify that a country is moving in the direction of higher AGWG in relative terms. Positive numbers signify the opposite. The point estimates are accompanied by the confidence intervals. See explanation under S1 and S2 Tables for the details of the methods compared. Data available at [http://grape.org.pl/data/gender-wage-gaps-around-eu-and-across-methods].

**Fig 5:**
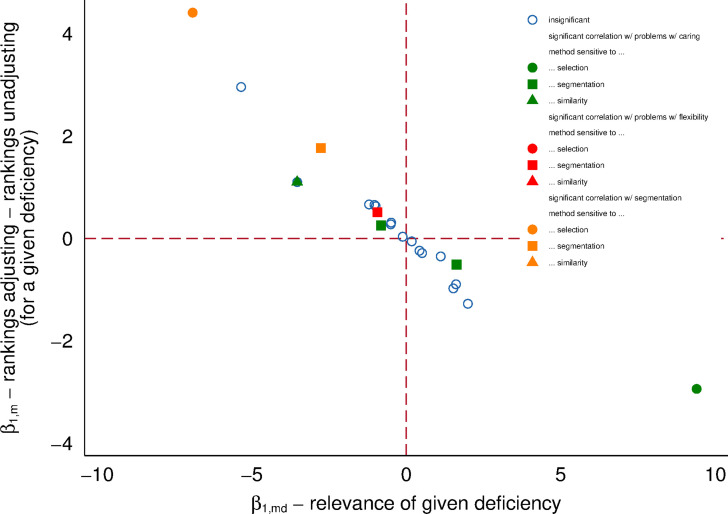
Correlations between rankings and labor market deficiencies. The horizontal axis denotes the strength of the correlation between given labor market deficiency and the rankings. The vertical axis denotes the difference in constants from Model (3) between the rankings based on a method with and the rankings without a given type of adjustment. We report all estimated coefficients from the model, and mark those which pass the bar of 15% significance. Methods sensitive to selection have to have selection correction (see Table 2). Methods sensitive to segmentation have to adjust for industry, occupation or firm size. Methods sensitive to the existence of similar men and women are non-parametric and semi-parametric methods from Table 2. Data available at [http://grape.org.pl/data/gender-wage-gaps-around-eu-and-across-methods].

We illustrate this point in two ways. First, in Fig 4, we portray that for some countries, a given feature of adjusting for obtaining the measures of AGWG changes the place in the ranking substantially. To provide a systematic overview of the dispersion in country rankings across methods and conditioning sets, we regress a ranking of a country from a given specification against a set of dummies characterizing given specification. Second, in Fig 5 we show that the changes in the ranking across methods can be explained by the sensitivity of decomposition methods to labor market deficiencies.

### 4.1. Systematic changes to rankings

Recall that for each country, we have 189 rankings stemming from the estimates of AGWG at the mean; in addition, we have 91 rankings for each country stemming from estimates of AGWG at each quartile. Each of those estimates is characterized by a method and set of covariates. We estimate the following two models. First, we estimate

$$\forall \text{​}c\text{​}\in countries\text{:}ranking_{m}=\beta_{0}+\beta_{1}functionalform+\beta_{2}selection+\beta_{3}CS+\delta_{1}occupation\text{+}\delta_{\text{2}}industry+\delta_{3}firmsize+\gamma WS+\in_{m},$$ (Model 1)

where *β*, *γ* and *δ* denote the estimated parameters, *functional form* denotes a dummy taking on the value of 1 if a given decomposition method has to assume a functional form of the wage determinants for both genders, *selection* denotes a dummy taking on a value of 1 if a given decomposition method corrects for selection into FTFY employment, *CS* is a dummy taking on a value of 1 if a given decomposition method restricts the sample to comparable men and women. In addition, the dummy variables *occupation*, *industry* and *firm size* denote dummies signifying if a given estimation included among the covariates occupation, industry and firm size, respectively. Finally, *WS* denotes a set of dummies for the counterfactual wage structure used in the decomposition (male, female, mixed, etc.). The second model is described by:

$$\forall \text{​}c\text{​}\in countries\text{:}ranking_{m}=\beta_{0}+\beta_{1}Q1+\beta_{2}Q3+\delta_{1}occupation\text{+}\delta_{\text{2}}industry+\delta_{3}firmsize+\gamma WS+\in_{m},$$ (Model 2)

where Q denotes quartiles at which decompositions underlying given ranking was obtained. The reference category for quartile is estimates at the median. Overall, Model 1 is based on 189 observations, and we estimate 23 such models, and Model 2 is based on 91 observations, and we estimate 23 such models. The estimated coefficients have a convenient interpretation because they summarize the change in ranking for a given country in rankings across methods. Note that with rankings as an explained variable, if one country moves towards equality, then another country has to move towards inequality. This implies that the parameters should add up to 0 for all the countries. Positive coefficients indicate that adjusting for a given aspect in the specification is associated with a higher ranking for a given country (i.e., more unjust inequality of wages across genders in the ranking, that is in relative terms). We report the estimates from Model 1 in S1 Table and results from Model 2 in S2 Table. For illustration purposes, we also report the obtained estimates in Fig 4: the left and the middle panel refer to Model 1, whereas the right panel refers to Model 2.

Clearly, the characteristic that changes the rankings the most is whether or not a method corrects for selection into FTFY employment. Countries change rankings by as much as ten positions up and down, *ceteris paribus*. The Netherlands and Austria are the countries in which the change is the most visible. Each of them moves almost ten positions down, that is towards greater gender wage inequality. Spain and Denmark also see their position worsens once we correct for selection. Some Eastern European countries see an improvement in their rankings. Lithuania is the most visible example, but hardly the only one. Slovenia, the Czech Republic, and Slovakia also belong to this group of countries.

Changes in the rankings defy simple country groupings. Beyond Eastern Europe, Sweden also observes improvement in its ranking. By geography and social welfare institutions, one would place Sweden with other Northern European countries, and yet it is the only where correcting for selection leads to higher rankings (lower relative inequality). By the same token, recent history and market orientation would lead to a classification of Poland with other Eastern European countries, and yet correction for full-time employment leads to deterioration in the ranking comparable to Western counterparts.

Restricting the analysis to men and women who share similar characteristics can move countries between two and three positions in the rankings: countries in Western Europe (Germany, Finland, France, and Italy) observe a fall in their position, whereas mostly Eastern European countries move towards higher equality.

The results also speak about the relative role of labor market segregation, as reflected in changes in the set of variables included in the controls. France and Finland are two countries where the inclusion of controls for industry and occupation leads to a relative improvement in unjustified inequality. Taken together, these results suggest that in these countries, women and men work in different industries and occupations. However, once this segregation is accommodated for in the estimation method, the scope of unjustified inequality appears smaller: access to jobs is unequal, but pay is relatively more equal for equal jobs.

Finally, the last panel of Fig 4 shows that some EU countries struggle with the deficiency of sticky floors and glass ceilings more than others. The ranking of Germany substantially worsens at the lower end of the income distribution, which suggests that the problem of sticky floors is relatively more acute in this country. The opposite is true of Austria, Finland, Hungary, and Poland, where the scope of unjustified inequality increases relatively more at the top of the income distribution.

### 4.2. Correlates of changes to rankings

The characterization portrayed in Fig 4 reveals that specific methods yield substantially higher rankings (more inequality) for specific countries. We argue that the reshuffling of the rankings stems from labor market deficiencies because some methods are more sensitive to a given type of deficiency. For example, methods that correct for selection into FTFY employment can speak of equality in access to full-time jobs and employment stability across genders. We further explore this line of reasoning by estimating the following models.

$$ranking_{i,m}=\beta_{0}+\beta_{1}method_{i,m}\times deficiency_{i}+\in_{i},$$ Model 3

where *i* denotes country, *m* denotes estimation method and × symbol denotes an interaction term, i.e., the base levels of both the *deficiency*_*i*_ variable and the *method*_*i*,*m*_ dummy as well as their interaction. In this notation, *β*_**1**_ is a vector of three parameters: *β*_**1**,*m*_ (which captures the difference in constants between rankings based on a specific *method*_*m*_ = 1 and rankings based on all other methods) *β*_**1**,*d*_ (which captures the correlation between rankings based on all methods except for *m* and deficiency) and *β*_**1**,*md*_ (which captures the additional correlation between the deficiencies and the rankings based on a specific *method*_*m*_ = 1). We estimate Model 3 as many times as there are combinations between method and deficiency in our sample (three deficiencies and seven methods, so 21 times in total) always on a sample of 23 countries. With variation coming only from dispersion across 23 countries we run the risk of underpowered correlations of *β*_0_ and *β*_1_, so we use 15% confidence intervals to establish, if the estimated coefficient is significantly different from zero.

We construct measures of potential difficulties faced by women in the labor market: the constraints on time imposed by caring functions (which we call: problems w/ caring), the constraints on demanded working time flexibility (which is naturally related but not equivalent and which we call problems with flexibility), and gender segmentation (which signifies strong segregation of genders across jobs and industries). The need to provide care for relatives (children or adults) is measured as a proportion of women who, potentially provide care (i.e., those who live with small children or dependent adults in a household) and who report that the need to provide care constraints their ability to be active in the labor market, seek work, work at all, or work full time. Second, we construct a measure of the lack of flexibility in the labor market. This variable measures the proportion of women that report insufficient flexibility in their employment contracts. Finally, we use data on occupation (two digit ISCO 08 codes) and industry (one digit NACE rev.2) to compute measures of labor market segmentation. We compute the Duncan Index of Dissimilarity [40]. The index has a straightforward interpretation: it indicates the fraction of workers who would need to change their jobs to obtain an equal employment structure across genders. Higher numbers are indicative of a more gender segmented segregated labor market. We report the value of each measure for each country in S3 Table together with a list of variables used to derive these measures. For ease of interpretation, when estimating Model 3, we normalize these three measures of deficiency.

Given that rankings have variation within each country, whereas labor market characteristics vary only between countries, we estimated Model 3 using multi-level regressions. This implies that the standard errors of *β*_1_ estimates adjust for the fact that the interaction term varies within the country, but measures of labor market deficiencies vary only across countries. Furthermore, we cluster standard errors at the country level. Notice that since rankings are obtained independently for each method, control set, and quartile, the regression is comparable to one, including fixed effects for the interaction of these elements. In other words, if one were to include controls for the variables presented in Fig 4 they would have all zero coefficients. The results are reported in Fig 5.

The interpretation of Fig 5 is straight forward, once the intuition behind Model 3 is explained. Consider the following thought experiment: take insufficient working time flexibility, WTF, as an example of a labor market deficiency. Further, take rankings based on methods which correct into FTFY employment, as an example of a method. Next, estimate a model of the form *g*_*i*_ = *γ*_0_ + *γ*_1_ *WTF*_*i*_ + *ϵ*_i_ *if* *method* ∈ *selection* *correction*, where *i* denotes country and the model is obtained from a multilevel regression and with clustered standard errors. Now, repeat the exercise with the following modification: $ranking_{i}={\tilde{\gamma }}_{0}+{\tilde{\gamma }}_{1}WTF_{i}+{\tilde{\epsilon }}_{i}\;if\;method\notin selection\;correction$. You can think of the parameters in Model 3 as $\beta_{1,md}=\gamma_{1}-{\tilde{\gamma }}_{1}$, and $\beta_{1,m}=\gamma_{0}-{\tilde{\gamma }}_{0}.$ Consequently the estimate *β*_**1**,*md*_ from Model 3 informs about the difference in slopes relating rankings (with and without selection correction in our example) and labor market deficiency (insufficient working time flexibility). The very existence of the slope between ranking of gender inequality and WTF, denoted in Model 3 by *β*_**1**,*d*_ is uninteresting, because it signifies in our example that on average countries with shorter supply of WTF tend to have higher gender inequality–an intuitive but not powerful observation. However, if *β*_**1**,*md*_ is significant, then clearly a given deficiency and a ranking based on a given method are *correlated through the sensitivity of the method*, and not merely through unequal societies having more unequal labor markets.

As portrayed in Fig 5, most interaction terms are not significant, which conforms with the expectations: if a method is not sensitive to detecting given type of labor market deficiency, correlation between ranking and this deficiency would not be stronger or weaker for this method than overall. However, of the 189 combinations between method and the set of the control variables, we expect some methods to be particularly sensitive to gendered selection into employment (denoted by circle), gendered segmentation of jobs (denoted by square) and avoiding the mistake of comparing the dissimilar workers (denoted by a triangle). For those of the 189 combinations, where one or more of those three conditions are fulfilled, one should expect *β*_**1**,*md*_ to be significant for the three measures of labor market deficiencies, as is the case. Most of the significant estimates fall in the fourth quadrant of Fig 5, where *β*_**1**,*m*_ is positive, and *β*_**1**,*md*_ is negative. The negative sign in *β*_**1**,*md*_ implies that ranks unadjusted for a given deficiency are downward biased where this deficiency is more prevalent. Once we account for this deficiency, rankings go up in countries where they were previously low, and fall where they used to be high. The positive sign of *β*_**1**,*m*_ is a mathematical consequence of the fact that all rankings–regardless of whether they are sensitive to a given deficiency or not–vary between 1 and 23. We also find two significant estimates in the second quadrant, (where *β*_**1**,*md*_ is positive, and *β*_**1**,*m*_ is negative), which implies the same downward bias as in the fourth quadrant.

In summary, Fig 5 demonstrates that the countries with relatively more constraints on primary care-givers to have a paid job tend to have lower unjustified inequality in wages, on average. Likewise, countries with more gender segmentation of jobs actually pay more equal for the jobs, where both men and women are employed–inequality is related to unequal access to many jobs, but in relative terms, pay is more equal for equal work. Thus, we make a case that exploiting rankings based on a broad variety of methods, differing in how sensitive they are to detecting various types of gender inequality, we one can inform policy debate about the type of labor market deficiencies which are–in relative terms–more of a policy issue in some countries than in others.

## 5. Conclusions

For policy-relevant reasons, inequality in many outcome variables such as wages, educational attainment, or exclusion is typically evaluated in a comparative perspective through rankings. Such rankings serve to identify potential policy priorities, evaluate policy changes, etc. While perfect equality may be unattainable for many outcome variables, countries may want to avoid lagging behind the reference countries with similar overall standards of living or aspirations. Such rationale lays the foundations for many international ranking efforts, including the examples of Gender Equality Index (for gender), the ranking of racial equality for the states of the USA, and many others. Formulation of such rankings is typically preceded by an intensive public debate on which issues to rank and how to formulate the ranking. Against this demand for rankings of inequality, the academic community provides a plethora of methods of obtaining the measures of *unjustified* inequality. None of the available methods is perfect in the sense that none is sensitive to all kinds of deficiencies. This would not matter if all countries were subject to the same deficiencies. However, if the countries differ in prevailing deficiencies, then “one method does not fit them all” and rankings based on any single method may be misguiding.

In this paper, we test empirically if this theory-based intuition delivers large or small discrepancies between the rankings of the 23 European countries for the case of adjusted gender wage gaps. For these economies, we show that rankings obtained from the same data depend crucially on the method used and its ability to highlight the deficiencies of the analyzed labor markets. Our focus on wages implies that we study a relatively broadly documented set of labor market deficiencies (access to labor market, segmentation, and differential effects along the distribution of wages). Studying gender wage gaps implies that we operate in a sphere of immensely developed applied econometrics, with a plethora of ways to adjust for differences in characteristics (unlike the case of other inequalities, such as entrepreneurship, access to credit or public goods, political activism, education, violence, etc., where the natural outcome measures are binary, count or categorical variables).

Our study provides two contributions. First, we show that the inherent features of a method may be useful in identifying the sources of inequality in a comparative perspective. Depending on the method, the set of covariates, the moment of the distribution, most of the countries can change their rank from the most equal to the most unequal. We also show that this variation of rankings correlates well with labor market deficiencies through the interaction of method and deficiency. The methods that a priori are more able to detect one type of inequality exhibit significant interactions with indirect proxies of this type of inequality. For example, the ranking in the case of Germany depends substantially on whether or not the estimation adjusts for labor market segregation and “sticky floors”. We infer that Germany may be characterized by having relatively less segmented labor markets than other EU countries and that the phenomenon of unjustified wage inequality concentrates at the lower and, to a lesser degree, upper parts of the earnings distribution. By contrast, in France, there appears to be substantially more scope for segmentation and the problem of gender wage inequality is relatively equally distributed along income. Comparative studies like ours may yield important policy implications for prioritizing interventions in the spirit proposed by [41].

Second, by exploiting a fixed selection of data sets across multiple estimation methods, quartiles of the wage distribution, and control variables, we show that no single method is equally reliable across differentiated origins of inequality. Hence, each ranking (and each underlying measure) will give a “false” premium to some countries and a “false” penalty to the other countries. It is easy to purposefully choose one specific ranking to give the impression that countries with a given type of deficiency are relatively more equal than they actually are. If a country has issues with segmentation, the country’s leaders may prefer a ranking based on a method that, while it is scientifically sound, is weak in identifying that particular deficiency. The way to mitigate potentially misguided implications of inadequate rankings is—as we show in this paper—to develop a broad array of rankings and study their changes to capture the relative severity of the deficiencies across countries that are being ranked. Much like GoogleWords, which operate in relative terms, one can interpret changes in positions across multiple rankings rather than focus on one selected indicator of inequality, potentially inadequate for a large part of studied countries. Our approach would be applicable to many alternative outcome measures, such as ranking equality in access to education, public service, health, poverty, and other policy-relevant contexts. Admittedly, our results apply to the same extent in a corporate context, where units or business lines are to be ranked for the purposes of compensation or setting output targets, all the more so if one attempts to account for structural differences between these units.

Our findings support several policy implications. First, rankings can be a useful policy and communication tool only if they reflect relevant inequality dimensions. This requires estimating many rankings across methods and covariates. Indeed, we show that the concern that a weakness of a given method to compute inequality may interact with the deficiencies of markets and institutions which generate this very inequality is not purely theoretical. Empirically, a ranking of a country varies by as much as ten ranks (out of 23).

Second and accordingly, rankings of gender wage inequality in Europe need to adjust for the ability to work, primarily work in full-time positions. We show that a key policy challenge in a cross-country perspective relates to working time flexibility and work-life balance. Not only do these indicators correlate well with the rankings of gender wage inequality, but methods that adjust for the ability to work, especially full-time, yield substantially different rankings to methods that fail to account for this dimension of gender inequality. Meanwhile, accounting for gender segregation into jobs or occupations, while potentially relevant for gender inequality in specific countries, does not lead to any major reshuffling of cross-country rankings. Finally, sticky floors or glass ceilings issues do not appear to be homogeneous across Europe: either or both of these issues prove to be relevant in some countries, but not in others. While this cross-country comparison reveals which countries need to prioritize policy interventions to address inequality at the bottom or the top of the income distribution, given the variation across countries, addressing inequality along the distribution does not appear to be a pan-European policy challenge.

## Supporting information

S1 File (ZIP)

S1 Table Drivers of country rankings–methods and control variables. (DOCX)

S2 Table Drivers of country rankings—AGWG at quartiles (relative to estimates at the mean). (DOCX)

S3 Table Measures of labor market deficiencies. (DOCX)
