# Supplementary material for: How rankings disguise gender inequality: A comparative analysis of cross-country gender equality rankings based on adjusted wage gaps
Source: PLoS One. 2020 Nov 4;15(11):e0241107. doi: 10.1371/journal.pone.0241107 (PMC7641444; doi:10.1371/journal.pone.0241107)
Supplement: S1 Table — (DOCX) [file pone.0241107.s002.docx]

S1 Table. Drivers of country rankings – methods and control variables.

|  | AT | BE | BG | CZ | DE | DK | EE | ES | FI | FR | GR | HU |
| --- | --- | --- | --- | --- | --- | --- | --- | --- | --- | --- | --- | --- |
| Occupation | 0.90*** | -0.31 | 0.62 | 2.70*** | 2.07*** | 0.18 | 0.01 | -0.56** | -2.92*** | -2.71*** | 0.4 | 1.89*** |
|  | [0.28] | [0.21] | [0.48] | [0.62] | [0.28] | [0.29] | [0.51] | [0.28] | [0.36] | [0.34] | [0.63] | [0.27] |
| Industry | -1.12*** | 0.52** | -2.03*** | -0.94 | -0.74*** | -0.60** | 0.84 | 1.43*** | -2.06*** | -0.96*** | 2.63*** | -0.19 |
|  | [0.28] | [0.21] | [0.48] | [0.62] | [0.28] | [0.29] | [0.51] | [0.28] | [0.36] | [0.34] | [0.63] | [0.27] |
| Firm controls | 0.03 | 0.28 | 0.11 | 1.09* | 0.07 | -0.47* | -2.12*** | -1.23*** | 1.10*** | -0.15 | -1.60** | -0.14 |
|  | [0.28] | [0.21] | [0.48] | [0.62] | [0.28] | [0.29] | [0.51] | [0.28] | [0.36] | [0.34] | [0.63] | [0.27] |
| Common Support | 0.66** | 0.52** | -2.89*** | -2.85*** | 2.44*** | 0.50* | -2.19*** | -2.22*** | 2.44*** | 3.03*** | 0.15 | 0.61** |
|  | [0.27] | [0.20] | [0.46] | [0.60] | [0.27] | [0.27] | [0.49] | [0.27] | [0.35] | [0.33] | [0.60] | [0.26] |
| Functional form | -2.65*** | -0.22 | -0.81 | 0.06 | 1.93*** | 0.61 | -0.37 | 0.53 | 2.74*** | 1.75** | -0.93 | -0.01 |
|  | [0.56] | [0.42] | [0.95] | [1.23] | [0.56] | [0.56] | [1.00] | [0.56] | [0.71] | [0.67] | [1.24] | [0.53] |
| Selection | 9.53*** | 3.69*** | -1.10** | -7.47*** | -1.07*** | 7.42*** | -2.92*** | 8.78*** | 3.19*** | 4.44*** | 0.66 | -1.40*** |
|  | [0.29] | [0.22] | [0.50] | [0.65] | [0.29] | [0.30] | [0.53] | [0.29] | [0.37] | [0.35] | [0.65] | [0.28] |
| Constant | 8.04*** | 2.51*** | 21.58*** | 21.19*** | 1.78*** | 3.90*** | 24.88*** | 12.57*** | 10.47*** | 5.75*** | 4.39*** | 11.90*** |
| Observations | 189 | 189 | 189 | 189 | 189 | 189 | 189 | 189 | 189 | 189 | 189 | 189 |
| R-squared | 0.86 | 0.64 | 0.28 | 0.51 | 0.52 | 0.8 | 0.29 | 0.86 | 0.63 | 0.66 | 0.12 | 0.35 |
|  | IT | LT | LV | NL | PL | PT | RO | SE | SI | SK | UK |  |
| Occupation | -1.46* | -0.19 | -1.24** | 0.63*** | 1.08*** | -0.58*** | 0.21 | -0.3 | 0.13 | 0.81 | -1.38*** |  |
|  | [0.77] | [0.16] | [0.53] | [0.24] | [0.20] | [0.22] | [0.32] | [0.33] | [0.18] | [0.53] | [0.23] |  |
| Industry | 3.16*** | 0.50*** | -3.22*** | 0.98*** | 2.58*** | 1.90*** | 2.00*** | -4.63*** | 0.17 | -0.11 | -0.1 |  |
|  | [0.77] | [0.16] | [0.53] | [0.24] | [0.20] | [0.22] | [0.32] | [0.33] | [0.18] | [0.53] | [0.23] |  |
| Firm controls | 2.02*** | -0.69*** | -0.56 | -0.13 | -0.27 | -0.23 | 0.26 | 0.85** | 0.22 | 0.06 | 1.49*** |  |
|  | [0.77] | [0.16] | [0.53] | [0.24] | [0.20] | [0.22] | [0.32] | [0.33] | [0.18] | [0.53] | [0.23] |  |
| Common Support | 3.76*** | 0.22 | -1.57*** | 0.59** | -0.09 | -0.72*** | 0.61** | 1.70*** | -1.00*** | -2.38*** | -1.35*** |  |
|  | [0.74] | [0.16] | [0.51] | [0.23] | [0.19] | [0.21] | [0.30] | [0.32] | [0.17] | [0.51] | [0.22] |  |
| Functional form | -0.13 | 0.45 | -1.44 | -0.16 | -2.41*** | 0.37 | 0.12 | 0.57 | 1.59*** | -0.37 | -1.24*** |  |
|  | [1.52] | [0.32] | [1.05] | [0.47] | [0.40] | [0.43] | [0.62] | [0.66] | [0.36] | [1.05] | [0.45] |  |
| Selection | 1.13 | -14.79*** | -5.62*** | 9.67*** | 4.86*** | 2.85*** | 0.87*** | -8.65*** | -10.16*** | -5.11*** | 1.20*** |  |
|  | [0.80] | [0.17] | [0.55] | [0.25] | [0.21] | [0.23] | [0.33] | [0.34] | [0.19] | [0.55] | [0.24] |  |
| Constant | 3.07** | 21.27*** | 24.61*** | -0.17 | 12.66*** | 16.22*** | 10.30*** | 12.42*** | 14.70*** | 19.23*** | 12.72*** |  |
| Observations | 189 | 189 | 189 | 189 | 189 | 189 | 189 | 189 | 189 | 189 | 189 |  |
| R-squared | 0.25 | 0.98 | 0.5 | 0.91 | 0.82 | 0.6 | 0.29 | 0.84 | 0.95 | 0.39 | 0.48 |  |

*Note:* Table displays results from an independent regression for every country, run on 189 estimates of adjusted gender wage gaps from various methods. Ranking position of the country is the dependent variable, with lower values identifying relatively more gender equal wages; whereas RHS variables are dummies, hence coefficients may be interpreted as shifters of the country ranking position. Data from EU-SILC 2013. ***, ** and * denote p*<*0.01, p*<*0.05, * p*<*0.1, respectively; t-statistics in parentheses. *Occupation*, *Industry* and *Firm control* denote dummies for specifications which adjust for occupation, control for industry and control for firm size, respectively. The *common support* dummy takes the value of 1 if method allows for estimating the AGWG only among similar individuals [31][.](#_bookmark48) The *functional form* dummy takes the value of 1 if the method is parametric, i.e. the results of decomposition depend on the functional form assumed [14–17,20–22]. *Selection* takes the value of 1 if the method corrects for selection into employment in computing AGWG [23]. Additional controls for counterfactual wage structure (male, female, mixed) are included and those results are available upon request. The base level are semi-parametric estimates [[32]](#_bookmark49) with no controls for industry, occupation and firm size and with no correction for selection.
