# Supplementary material for: How rankings disguise gender inequality: A comparative analysis of cross-country gender equality rankings based on adjusted wage gaps
Source: PLoS One. 2020 Nov 4;15(11):e0241107. doi: 10.1371/journal.pone.0241107 (PMC7641444; doi:10.1371/journal.pone.0241107)
Supplement: S2 Table — (DOCX) [file pone.0241107.s003.docx]

**S2 Table. Drivers of country rankings - AGWG at quartiles (relative to estimates at the mean).**

|  | AT | BE | BG | CZ | DE | DK | EE | ES | FI | FR | GR | HU |
| --- | --- | --- | --- | --- | --- | --- | --- | --- | --- | --- | --- | --- |
| Q25 | -0.84* | 1.08*** | -1.34*** | 4.68*** | 5.56*** | -1.13*** | -0.2 | -1.05*** | -1.64*** | -1.25*** | -1.41*** | -5.14*** |
|  | [0.43] | [0.38] | [0.31] | [0.37] | [0.37] | [0.22] | [0.35] | [0.35] | [0.41] | [0.16] | [0.46] | [0.25] |
| Q75 | 2.85*** | -2.52*** | 1.45*** | -0.27 | 2.23*** | 1.03*** | -0.22 | -5.21*** | 5.48*** | -0.2 | -3.10*** | 3.55*** |
|  | [0.43] | [0.38] | [0.31] | [0.37] | [0.37] | [0.22] | [0.35] | [0.35] | [0.41] | [0.16] | [0.46] | [0.25] |
| Constant | 8.07*** | 5.29*** | 19.74*** | 17.60*** | 1.76*** | 3.16*** | 23.24*** | 14.83*** | 13.27*** | 5.56*** | 7.39*** | 13.78*** |
| Observations | 273 | 273 | 273 | 273 | 273 | 273 | 273 | 273 | 273 | 273 | 273 | 273 |
| R-squared | 0.34 | 0.28 | 0.31 | 0.49 | 0.6 | 0.32 | 0.05 | 0.53 | 0.66 | 0.55 | 0.2 | 0.83 |
|  |  |  |  |  |  |  |  |  |  |  |  |  |
|  | IT | LT | LV | NL | PL | PT | RO | SE | SI | SK | UK |  |
| Q25 | -0.37 | 1.40** | -3.52*** | -0.46 | -1.51*** | -1.66*** | 3.27*** | 2.42*** | 1.15*** | 0.42 | 1.54*** |  |
|  | [0.34] | [0.58] | [0.36] | [0.64] | [0.25] | [0.40] | [0.27] | [0.33] | [0.44] | [0.34] | [0.22] |  |
| Q75 | 2.15*** | -2.18*** | -1.13*** | 0.57 | 2.35*** | -2.87*** | 1.69*** | 2.46*** | -4.58*** | -3.11*** | -0.44** |  |
|  | [0.34] | [0.58] | [0.36] | [0.64] | [0.25] | [0.40] | [0.27] | [0.33] | [0.44] | [0.34] | [0.22] |  |
| Constant | 5.02*** | 20.74*** | 21.78*** | 1.25 | 13.00*** | 18.49*** | 12.18*** | 9.01*** | 11.72*** | 18.00*** | 11.13*** |  |
| Observations | 273 | 273 | 273 | 273 | 273 | 273 | 273 | 273 | 273 | 273 | 273 |  |
| R-squared | 0.37 | 0.15 | 0.34 | 0.05 | 0.6 | 0.25 | 0.46 | 0.65 | 0.46 | 0.34 | 0.35 |  |

*Note:* Table displays results from an independent regression for every country, run on 273 estimates of adjusted gender wage gaps from various methods at the 25th, 50th, 75th percentile [26, 28, 32]. The median is the reference category for percentiles. Estimates include also covariates for counterfactual wage structure, and the set of dependent variables used in the decomposition. Ranking position of the country is the dependent variable, with lower values identifying relatively more gender equal wages; RHS variables are dummies, hence coefficients may be interpreted as shifters of the country ranking position. Data from EU-SILC 2013. ***, ** and * denote p*<*0.01, p*<*0.05, * p*<*0.1, respectively; t-statistics in parentheses. Q*25, Q50 and Q75 percentile* take the value of 1 if the AGWG was estimated at those quartiles and zero otherwise. The estimates at the mean constitute the base category.
