# Supplementary material for: How rankings disguise gender inequality: A comparative analysis of cross-country gender equality rankings based on adjusted wage gaps
Source: PLoS One. 2020 Nov 4;15(11):e0241107. doi: 10.1371/journal.pone.0241107 (PMC7641444; doi:10.1371/journal.pone.0241107)
Supplement: S3 Table — (DOCX) [file pone.0241107.s004.docx]

**S3 Table. Measures of labor market deficiencies.**

| Country | Problems w/caring | Problems w/flexibility | Segmentation |
| --- | --- | --- | --- |
| AT | 0.089 | 0.814 | 0.523 |
| BE | 0.035 | 0.758 | 0.523 |
| BG | 0.002 | 0.906 | 0.570 |
| CZ | 0.024 | 0.848 | 0.578 |
| DE | 0.093 | 0.868 | 0.509 |
| DK | 0.006 | 0.749 | 0.500 |
| EE | 0.025 | 0.874 | 0.602 |
| ES | 0.064 | 0.851 | 0.519 |
| FI | 0.022 | 0.819 | 0.586 |
| FR | 0.049 | 0.794 | 0.512 |
| GR | 0.033 | 0.691 | 0.407 |
| HU | 0.024 | 0.859 | 0.555 |
| IT | 0.042 | 0.823 | 0.492 |
| LT | 0.013 | 0.896 | 0.569 |
| LV | 0.014 | 0.922 | 0.601 |
| NL | 0.069 | 0.871 | 0.529 |
| PL | 0.020 | 0.768 | 0.522 |
| PT | 0.022 | 0.827 | 0.520 |
| RO | 0.018 | 0.747 | 0.469 |
| SE | 0.002 | 0.776 | 0.509 |
| SI | n.a. | 0.784 | 0.525 |
| SK | 0.005 | 0.845 | 0.593 |
| UK | 0.002 | 0.746 | 0.513 |

*Note*: Variables derived from the Labor Force Survey of the EU.

Problems w/caring is the proportion of women living with small children or dependent adults in a household and who report that the need to provide care constraints their ability to be active in the labor market. For workers who left the labor market, we use the variable –leavereas– (which reports reasons for leaving the labor market) and–seekreas– (which reports the reasons for not searching employment) to indicate caring constraints. For workers in part-time jobs we employ –ftptreas– (which reports reasons for working part-time). Finally, we also utilize –needcare– and –availreas– (where responders report need for caring facilities and whether or not these needs are satisfied, respectively). Each population is considered independently.

Problems w/flexibility measures the proportion of women that report insufficient flexibility in their employment contracts (actual or offered). . Workers report if they work from home (variable –homewk–), and if they would prefer to work fewer hours (variable –hwwish– smaller than –hours–) or more hours (variable –wishmore–). In addition, we use information about reasons for looking a different job (variable –lookreas– where individuals report that they look for a job with a different number of hours as the current one or different working time arrangements) as well as the type of job they seek (variable –seektype– where individuals report that they search part-time work).

Segmentation is the Duncan index of dissimilarity computed over jobs (occupation in a given industry). The index is defined as $segmentation=\sum_{i\in I} \sum_{o\in O} |\frac{m_{i,o}}{M}-\frac{w_{i,o}}{W}|$, where $m_{i,o}$ is the number of men working in industry $i$ and occupation $o$, and $M$ is the total number of men in the sample $w_{i,o}$, and $W$ are defined analogously for women. Higher values indicate more segmented markets.

*Comment*: These indicators are not intended as a perfect “measure” of the three specific deficiencies. These deficiencies are but an example of labor market characteristics that are difficult to capture (unlike behaviors, preferences or constraints are not directly observable) and for which a consensus measures do not exist. We consider them proxies, which should generally correlate with the deficiencies that they are intended to describe.
